# Photogrammetry in Dentistry: From Digital Impressions to Clinical Workflows

**DOI:** 10.7759/cureus.117457

**Published:** 2026-10-04

**Authors:** Akshay Katara, P. Nihar, Anand P Bhalodi, Ajay Kumar Dogra, Mohit M Galani, Vikas Singh, Anukriti Kumari, Ritik Kashwani

**Affiliations:** 1 Department of Dentistry, Omni Family Health, Bakersfield, USA; 2 Department of Conservative Dentistry and Endodontics, CKS Theja Dental College, Tirupati, IND; 3 Department of Dentistry, Family HealthCare Network, Visalia, USA; 4 Department of Healthcare Management, University Institute of Applied Management Sciences (UIAMS), Chandigarh, IND; 5 Department of Dentistry, All India Institute of Medical Sciences, Rajkot, IND; 6 Department of Public Health Dentistry, Teerthanker Mahaveer Dental College and Research Centre, Moradabad, IND; 7 Department of Oral Medicine and Radiology, School of Dental Sciences, Sharda University, Greater Noida, IND; 8 Department of Dental Research Cell, Dr. D. Y. Patil Dental College and Hospital, Dr. D. Y. Patil Vidyapeeth (Deemed to be University), Pune, IND

**Keywords:** cad/cam, digital dentistry, digital impressions, implant dentistry, intraoral scanning, photogrammetry, stereophotogrammetry

## Abstract

A three-dimensional digital technology called photogrammetry is gaining popularity and is becoming a valuable tool in dentistry, especially when it comes to documenting the position of dental implants and creating complex digital workflows. This narrative review discusses the principles, methods, applications, accuracy, benefits, limitations, and current clinical applications of photogrammetry in dental practice, with a specific focus on implant-supported rehabilitation and complete-arch digital impressions. The use of conventional photogrammetry, stereophotogrammetry, image acquisition, three-dimensional coordinate reconstruction, intraoral scanning, computer-aided design/computer-aided manufacturing (CAD/CAM), and digital implant workflows is discussed. There is currently evidence that photogrammetric methods can be used to record complete-arch implant position with high trueness and precision, and in certain experimental and clinical trials, lower deviations in 3D and angular measurements were reported than with intraoral scanning. But it is hard to compare results across studies because of variations in the types of photogrammetric systems, acquisition protocols, designs of reference markers, software algorithms, distributions of implants, and methods of measurements. In addition, the intraoral surface anatomy and soft-tissue morphology captured routinely via photogrammetry are not as complete as what can be captured by intraoral scanning, and so a combined workflow may collect more comprehensive digital information. As the technology advances, photogrammetry is shifting away from the role of simply recording the position of implants and moving towards utilizing it as a part of digital dentistry, with possibilities in prosthodontics, implant dentistry, facial scanning, maxillofacial rehabilitation, and computer-assisted treatment planning.

## Introduction and background

Digital technology has revolutionized diagnostic, restorative, implant and prosthodontic workflows. While conventional impressions are clinically proven, they still require several physical processes: impression materials, impression trays, impressions, laboratory scanners, and manual transfer of information from a 3D impression [1]. In recent years, digital impression systems have become increasingly significant as they can directly create 3D data that can be used with computer-aided design and computer-aided manufacturing (CAD/CAM), improve clinical-laboratory communication, and possibly streamline restorative processes [2]. For a whole edentulous arch with several implants, however, it is difficult to ensure dimensional accuracy, as small deviations in position can add up during the process of acquiring images and stitching them together [3].

Photogrammetry is a measurement method for 3D purposes that involves computing the spatial position of objects from the photograms or optical images. It has also been widely used in dentistry, where a number of dedicated stereophotogrammetric systems have been developed that are able to record the 3D position of more than one implant scan body or photogrammetric marker [4]. Stereophotogrammetry (SPG) primarily measures the spatial relationships between certain set of reference markers, while conventional intraoral scanners (IOSs) are used to sequentially collect surface information and then combine the images. In a completely edentulous arch, where there are fewer anatomical landmarks and greater distances between teeth, this distinction can become important, potentially affecting the stitching (attachment) process of IOS systems [5].

General accuracy of a digital impression is usually measured by trueness and precision of the impression. The term “trueness” is used to quantify the agreement between a set of measurements and a reference set of measurements, whereas the term “precision” is used to quantify the agreement between a set of repeated measurements [6]. These parameters in implant dentistry can be assessed by linear deviations, 3D deviations, angular deviations, surface deviations, or inter-implant-distance deviations [7]. The clinical relevance is related to the ability to recreate the true three-dimensional location of implants and then manufacture prostheses with an acceptable passive fit. Positioning errors of implants can be a factor in the misfit of the prosthetic and could affect mechanical or biological complications with time [8]. Assessment of implant impression accuracy is therefore not limited to merely visually evaluating whether a digital scan is similar to the oral anatomy, but also needs to be quantified by evaluating the spatial relationship between the implant components [9].

Initial research on digital implant impressions showed critical issues for scanning multiple implants on an edentulous arch [10]. Follow-up systematic reviews revealed significant differences in the accuracy of full-arch digital implant impressions depending on the scanner type, scan strategy, distance between the scans, the characteristics of the scans, the operator, and other methodological factors [11]. Zhang et al. (2021) found some significant variability between different studies for linear and angular deviations for full-arch implant impressions, which can be a problem when the area is edentulous [12].

Some of these limitations have been tried to be overcome by the advent of photogrammetry. For dedicated SPG systems, the estimated implant positions are obtained by the use of calibrated cameras and reference markers, which does not require to continuously reconstruct the entire edentulous surface [13]. This may minimize the accumulated stitching errors and offer a more direct way to digitize the implant positions. Hussein's systematic review found that, at the time, there was a lot of variation in the quality and quantity of the evidence regarding the use of photogrammetry in implant dentistry, but this technology could be a useful tool for documenting implant position and facilitating digital implantology [14].

This comparative evidence has then given some further insight into the possible benefits of photogrammetry. SPG showed a lower 3D and angular deviation in a prospective study where both implant positions and number did not have a significant impact on its accuracy when compared with IOS [15]. 

In addition to implant position recording, other digital tools have the potential to incorporate IOS, facial scanning, CAD/CAM, computer-assisted implant planning, and virtual prosthetic planning. This integration may enable the use of each digital data to complement each other; precise coordinates to the implant provided by the photogrammetry, and surface morphology and soft-tissue data from the IOS [16]. This separation is clinically significant since the use of photogrammetry does not imply it is a substitute for IOS; it could have a different function and be complementary to it in an integrated digital workflow. Recent comparative research has thus shifted its attention increasingly to the co-combination of these technologies instead of just assessing the superiority of a single system [17].

Although all these developments have occurred, a number of issues remain. Comparisons between studies are difficult due to the differences in the photogrammetric devices, marker design, camera configuration, image acquisition protocol, software algorithm, implant distribution, technique used by the operator and reference standards [18]. Additionally, most evidence is of laboratory origin, and clinically relevant factors like implant fit, chairside modification, biological complications, long-term maintenance of implants, and patient outcomes are not sufficiently studied [19].

The aim of this narrative review is to overview the history of photogrammetry in dentistry, explain the principles and digital workflow of this discipline, and critically discuss its uses, accuracy, benefits, limitations, and new potential clinical applications. Special attention is given to implant dentistry, complete-arch digital impressions and the opportunities that arise when using photogrammetry alongside state-of-the-art CAD/CAM, intraoral scanning, and computer-assisted workflows in dentistry. To establish the clinical value-added of photogrammetry and the need for more studies to validate its use is imperative.

## Review

A narrative literature search was conducted using PubMed/MEDLINE, Scopus, Web of Science, and Google Scholar to identify relevant publications on photogrammetry in dentistry, with particular emphasis on implant position recording, complete-arch digital impressions, stereophotogrammetry, intraoral scanning, and integrated digital workflows. The search included studies published from database inception through September 2026. Search terms included combinations of “photogrammetry,” “stereophotogrammetry,” “intraoral photogrammetry,” “dental implant,” “complete-arch implant,” “digital impression,” “intraoral scanner,” “implant accuracy,” “trueness,” “precision,” and “CAD/CAM.” Reference lists of relevant systematic reviews and key primary studies were also screened to identify additional publications. Studies were considered relevant when they evaluated the accuracy, clinical application, workflow integration, advantages, limitations, or emerging applications of photogrammetry in dentistry. Priority was given to systematic reviews, meta-analyses, comparative in vitro and in vivo studies, and recent clinical or technological studies. Studies that were not directly related to dental photogrammetry or did not address the objectives of this review were excluded. Because this was a narrative review, the identified evidence was qualitatively synthesized according to study design, application, measurement parameters, comparative findings, and clinical relevance rather than subjected to a formal meta-analysis.

Introduction to photogrammetry and its development in dentistry

Photogrammetry is an optical three-dimensional measurement method that uses the spatial coordinates of objects within a photograph to reconstruct them. In dentistry, its growth has paralleled the growing need for precise digital transfer of implant location to the CAD/CAM workflow [20]. Traditional impressions reconstruct implant position indirectly using materials and casts, while photogrammetry captures the three-dimensional relationship between dedicated reference markers placed on the implants. This technique is especially important for complete-arch implant rehabilitation, as errors can accumulate during intraoral scanning with conventional intraoral scanners, potentially compromising the accuracy of the final dataset [21].

Stereophotogrammetry (SPG) is a photogrammetric technique that records the photogrammetric markers simultaneously from multiple cameras that are calibrated together. Images thus obtained are then processed on a computer to determine the spatial coordinates of each marker [22]. Unlike intraoral scanners (IOSs), SPG does not selectively reconstruct the entire intraoral surface but rather collects data of the position of the implants. Thus, it will reduce the chance of errors associated with stitching in long edentulous spans [23].

Applications in implant dentistry

The most common clinical use of photogrammetry is the digital impression of several implants, especially when the patient is edentulous. Properly recording implant positions is crucial for the design of fixed dental prostheses on implants, full-arch rehabilitation, and immediate loading restorations. High trueness and precision have been shown in studies showing that multiple implants can be recorded with high trueness and precision, and some studies have reported lower angular and 3D deviations than IOS [24].

In addition to this, PC-based photogrammetry software can be integrated into an IOS solution. In these workflows, SPG delivers precise implant positions, while IOS provides the surrounding hard- and soft-tissue morphology. The datasets can then be combined in CAD software to assist in prosthetic design. This complementary use may help overcome the limitations of either technology used alone [25]. Table 1 summarizes the major dental applications of photogrammetry.

**Table 1:** Major dental applications of photogrammetry. IOS: intraoral scanner; CAD/CAM: computer-aided design and computer-aided manufacturing.

Application	Primary purpose	Clinical relevance
Complete-arch implant impressions [4,13,22]	Record implant coordinates	Full-arch fixed prostheses
Multiple-implant rehabilitation [4,13,22]	Transfer spatial implant relationships	CAD/CAM prosthetic fabrication
Immediate-loading workflows [16,25]	Rapid digital implant transfer	Reduced laboratory turnaround
Implant prosthetic verification [22]	Assess spatial accuracy	Prosthesis fit evaluation
Facial photogrammetry [17]	Capture three-dimensional facial morphology	Esthetic and maxillofacial planning
Combined IOS-photogrammetry [16,25]	Merge implant and surface datasets	Comprehensive digital workflow

Accuracy and measurement parameters

The most important difference is that SPG does not require continuous surface stitching to establish the implant relationship. Several complete-arch implant studies have reported differences in three-dimensional and angular deviations between IOS and SPG, with results varying according to the scanner, photogrammetric system, implant distribution, and measurement methodology [26,27]. However, IOS provides surface anatomy and soft-tissue morphology that are not routinely captured by SPG. Thus, fusion of both technologies could offer a more comprehensive digital dataset [28]. A comparison of the principal characteristics of photogrammetry and IOS is presented in Table 2.

**Table 2:** Photogrammetry versus intraoral scanning. CAD/CAM: computer-aided design and computer-aided manufacturing.

Parameter	Photogrammetry	Intraoral scanning
Primary dataset [4,13,22,27]	Implant/reference-marker coordinates	Surface morphology
Full-arch implant accuracy [15,21,22,29]	Generally high	Variable
Stitching dependence [13,21,29]	Minimal for implant coordinates	Relatively high
Soft-tissue recording [16,25]	Limited	Available
Implant coordinate recording [4,13,22]	Major strength	Device-dependent
CAD/CAM integration [16,25]	High	High
Complete digital arch information [16,25]	Usually requires complementary scanning	Directly available
Clinical workflow [16,25]	Specialized equipment	Increasingly routine

Comparison with intraoral scanning

Because of its ability to directly acquire a 3D surface without traditional impression techniques, IOS is a common part of digital dentistry today. However, there are some challenges to scanning the entire arch. The lack of stable anatomical landmarks in edentulous patients can impact the reliability of image stitching, and longer scans and greater distances between implants can add to cumulative deviations [29]. The most important difference is that SPG is not surface-stitched continuously for the establishment of implant relationships. Several complete-arch implant studies have reported lower three-dimensional and angular deviations with SPG compared with IOS, although results vary according to the scanning system, photogrammetric device, implant distribution, and measurement methodology [22,30]. However, IOS information is not routinely collected by SPG, especially detailed surface anatomy and soft-tissue morphology. Thus, a fusion of both technologies could offer a more comprehensive digital dataset.

Factors influencing photogrammetric accuracy

The performance of the photogrammetry could be affected by several factors. Many factors can influence the measurement results, including the number and spread of implants, the angle of the implants, the distance between implants, the geometry of the markers, the camera calibration, the image acquisition, environmental factors, software algorithms, and how the patient handles the device [31]. Reference-marker design is particularly relevant as markers are the geometric information that is used for spatial reconstruction.

Accuracy may change with the distribution of implants. A large spatial reference frame can be obtained with widely distributed implants, but at the same time, the technical requirements of the imaging process may be higher. It may be more difficult to position markers and images with angulated implants or limited access. Therefore, standardized acquisition protocols are needed before standardized methods of photogrammetry can be considered interchangeable with other impression techniques [32].

Integration with digital dentistry

The potential of photogrammetry is not that of replacing current digital technologies, but rather to complement them. In a combined workflow, precise implant coordinates can be obtained using photogrammetry; intraoral surfaces can be captured using IOS; and extraoral morphology can be captured using facial scanning. These datasets can then be combined in CAD to plan prosthetics and esthetics [33].

Data processing could be further enhanced by artificial intelligence and automated image-registration algorithms, further decreasing operator dependence. Cloud-based workflows may also enable better communication among dental offices, labs, and manufacturers. But these technologies need clinical validation before they can be widely used for all indications [34].

Advantages and limitations

The key benefits of using photogrammetry are accurate recording of implant position, reduced reliance on full-arch image stitching, rapid digital data collection, and compatibility with CAD/CAM workflows [35]. However, photogrammetry also has limitations, including equipment costs, the need for specialized training, limited capture of complete intraoral anatomy, and dependence on an additional IOS or other scanning technique when surface and soft-tissue information is required [36]. Table 3 summarizes the principal advantages and limitations of photogrammetry.

**Table 3:** Advantages and limitations of photogrammetry. IOS: intraoral scanner; CAD/CAM: computer-aided design and computer-aided manufacturing.

Advantages	Limitations
High implant-position accuracy [27,29]	Specialized equipment
Reduced stitching-related error [13,21,29]	Additional IOS may be required
Rapid acquisition [36]	Equipment cost
Suitable for complete-arch implant cases [13,22,27]	Limited soft-tissue information
CAD/CAM compatibility [16,25]	Technique-sensitive marker positioning
Reproducible implant coordinates [27]	Limited long-term clinical evidence

Current evidence and future perspectives

Systematic reviews and meta-analyses have recently shown that the accuracy of the complete-arch implant impression using photogrammetry can be extremely high, and that for some accuracy parameters, such as the three-dimensional and angular measurements of implant position, the method may have superior results compared to the IOS [22,37,38]. However, there is still significant inter-device, inter-standard, inter-implant, and inter-method variability.

Standardized accuracy protocols, prospective clinical trials, clinically meaningful prosthetic-fit outcomes, cost-effectiveness assessment, and long-term evaluations should, therefore, be the focus of future research. Despite the apparent rivalry between photogrammetry and IOS, recent studies indicate that they can be studied in relation to each other in integrated digital workflows [16,25,38].

Photogrammetry is a significant development in digital dentistry with the best evidence currently used in complete-arch implant position recording. Further development, integration with IOS and CAD/CAM, and the inclusion of automated computational technologies could expand its usage in a variety of implant prosthodontics, restorative dentistry, and maxillofacial rehabilitation applications [39].

## Conclusions

Photogrammetry is a valuable addition to modern digital dentistry, especially for precisely recording implant positions in complete-arch rehabilitation. In certain clinical situations, it offers important advantages in reducing cumulative stitching errors and improving the reproducibility of implant coordinates in space compared with conventional digital impression methods. Photogrammetry, however, does not provide all intraoral structures and therefore might be most useful when combined with intraoral scanning and CAD/CAM technologies. Direct comparisons across studies are not currently possible due to differences in equipment, acquisition protocols, reference markers, implant configuration, and measurement methods. Additional clinical studies with carefully designed clinical trials and standardized criteria for accuracy are needed to determine definitive clinical thresholds and long-term outcomes. Photogrammetry, combined with intraoral scanning, AI, and automated CAD/CAM workflows, could further broaden its applications in terms of more precise, efficient, and comprehensive digital dental rehabilitation solutions.
